# Impact of Neighborhood Socioeconomic Trajectories on Gastrointestinal Cancer Care: A SEER-Medicare Analysis

**DOI:** 10.1245/s10434-025-17764-1

**Published:** 2025-07-08

**Authors:** Mujtaba Khalil, Selamawit Woldesenbet, Shreya Shaw, Abdullah Altaf, Shahzaib Zindani, Zayed Rashid, Razeen Thammachack, Syed Husain, Timothy M. Pawlik

**Affiliations:** Department of Surgery, The Ohio State University Wexner Medical Center and James Comprehensive Cancer Center, Columbus, OH USA

**Keywords:** Redlining, Surgical outcomes, Textbook outcomes, Social determinants of health

## Abstract

**Background:**

Historical discriminatory policies, such as residential redlining, along with current socioeconomic status, may impact gastrointestinal (GI) cancer care. We sought to investigate how evolving neighborhood characteristics impact the diagnosis and treatment of GI cancer.

**Patients and Methods:**

Individuals who were diagnosed with GI cancer were identified using the Surveillance Epidemiology and End Results (SEER)-Medicare linked database. Neighborhood socioeconomic trajectories were determined using historical redlining grades and contemporary social vulnerability index scores. These trajectories were categorized as advantaged stable (chronically affluent neighborhoods), advantaged reduced (neighborhoods with declining affluence), disadvantaged stable (neighborhoods with chronic deprivation), and disadvantaged reduced (neighborhoods with declining deprivation). Multivariable regression was utilized to examine the association between neighborhood trajectory and stage at diagnosis, cancer-directed treatment, and surgical outcomes.

**Results:**

Among 15,118 individuals, 30.5% (*n* = 4608) resided in advantaged stable neighborhoods, 44.5% (*n* = 6727) in disadvantaged reduced neighborhoods, 2.96% (*n* = 448) in advantaged reduced neighborhoods, and 22.1% (*n* = 3335) in disadvantaged stable neighborhoods. Of note, individuals living in disadvantaged stable neighborhoods were less likely to undergo surgery (55.8% vs. 59.2%), receive chemotherapy (56.7% vs. 60.3%), and achieve a textbook outcome (TO) following surgery (41.3% vs. 51.3%) (all *p* < 0.001). On multivariable analyses, individuals living in disadvantaged stable neighborhoods had higher odds of being diagnosed at an advanced stage (OR 1.29, 95% CI 1.18–2.42) and lower odds of receiving chemotherapy (OR 0.67, 95% CI 0.58–0.76) and achieving a TO (OR 0.68, 95% CI 0.59–0.77).

**Conclusions:**

Individuals living in disadvantaged stable neighborhoods have advanced stages at diagnosis and experience poorer surgical outcomes. There is an urgent need for targeted interventions and policies to address structural inequities and ensure health equity.

Disparities in access to healthcare and health outcomes are well documented, with people of color and low-income individuals experiencing suboptimal results.^1,2^ These disparities stem from various levels of oppression and racism, including structural, interpersonal, and intrapersonal factors.^3^ Consequently, there is a growing emphasis on understanding social determinants of health (SDoH) and leveraging these factors to improve health outcomes.^3^ Previous studies have focused, however, solely on historical or current neighborhood characteristics, often overlooking the dynamic ways in which neighborhoods influence health.^3,4^ For example, affluent neighborhoods today may have histories of wealth accumulation or recent financial influxes due to development, which can displace lower-income residents.^5^ These evolving dynamics shape the structures, systems, and interpersonal interactions within communities, underscoring the need for a comprehensive understanding that transcends static historical or current contexts.^6^

Residential redlining has significantly shaped socioeconomic conditions in the past.^5,7^ Established in the 1930s by the Home Owners’ Loan Corporation (HOLC), this policy aimed to refinance home mortgages and classify neighborhoods on the basis of perceived risks to mortgage security.^7^ Of note, neighborhood classifications were influenced by factors such as housing conditions, transportation access, proximity to amenities, and racial/ethnic composition.^3^ Neighborhoods primarily inhabited by minority populations were often labeled “hazardous” and marked in red on HOLC maps.^3^ This designation led to economic disinvestment in these communities, worsening barriers to quality education, employment, income mobility, and healthcare access.^3,5^ A contemporary measure of socioeconomic conditions is the Social Vulnerability Index (SVI), developed by the Centers for Disease Control and Prevention (CDC).^8^ The SVI is a composite score derived from 18 population-level factors, including poverty, unemployment, transportation, housing, and education levels.^8^ This index identifies communities that may be particularly vulnerable to external stressors such as natural disasters, public health emergencies, or economic shifts.^8^

Given the dynamic nature of socioeconomic status, it is essential to consider both current and historical factors to fully understand their impact on healthcare outcomes.^6^ Nonetheless, previous studies have often focused exclusively on either historical or current neighborhood characteristics, neglecting how neighborhood socioeconomic status evolves over time.^4–6^ Therefore, the current study sought to utilize trajectory modeling to investigate the effects of residential redlining and SVI on disease stage at diagnosis, cancer-directed treatment, and surgical outcomes.

## Patients and Methods

### Data Source, Study Population, and Cohort Selection

The Surveillance Epidemiology and End Results (SEER)-Medicare linked database was queried using the International Classification of Diseases for Oncology (ICD-O-3) and the World Health Organization (WHO) 2008 SEER site recodes to identify patients diagnosed with gastrointestinal (GI) cancers. SEER database collects data on cancer incidence from 18 registries across 15 states, accounting for roughly 30% of the U.S. population.^9^ By matching 93% of Medicare beneficiary medical claims to individuals in the SEER registries, the national Medicare health insurance program covers 97% of people aged 65 and older with cancer.^9^ The study included patients diagnosed with hepatic, biliary tract, pancreatic, colon, or rectal cancer between 2007 and 2019. Additionally, to be included in the study, patients had to maintain continuous enrollment in Medicare Parts A and B for at least a year before and after the diagnosis of cancer. Patients diagnosed with GI cancer solely on the basis of autopsy or death certificate, as well as individuals who were younger than 66 years, had missing information, or presented with multiple primary cancer sites, were excluded. The Institutional Review Board at Ohio State University approved this study and waived the requirement for informed consent because the data were limited.

### Exposure

The primary exposure was neighborhood trajectory. Each patient’s area of residence, identified at the census tract level, was assigned a HOLC redlining grade and SVI score. HOLC redlining grades were obtained from the Mapping Inequality: Redlining in New Deal America project, while data on SVI was sourced from the CDC.^8,10^ HOLC redlining grades include A, B, C, and D.^3^ Neighborhoods rated as “A” were considered the “best” for investment, characterized by predominantly white, middle- to upper-income residents, well-maintained housing, and low mortgage default risk.^3^ Grade “B” areas were considered “still desirable,” Grade “C” areas were labeled “definitely declining,” and Grade “D” areas were classified as “hazardous,” predominantly inhabited by racial and ethnic minorities, suffering from significant economic decline, and viewed as the highest risk for investment.^3^ SVI was dichotomized into low and high vulnerability.

To evaluate changes in neighborhoods from historic HOLC grades to the current degree of vulnerability, neighborhood trajectories were calculated. Neighborhood trajectories were categorized as: “advantaged stable” for census tracts with HOLC grades A and B and low SVI; “advantaged reduced” for HOLC grades A and B and high SVI; “disadvantaged reduced” for HOLC grades C and D and low SVI; and “disadvantaged stable” for HOLC grades C and D and high SVI.

### Covariates and Outcomes of Interest

Baseline covariates included patient age, sex, race/ethnicity (categorized as white, Black, Hispanic, or other [other category included Asian, American Indian, and Alaska Native]), Charlson comorbidity index (CCI), cancer site, disease stage, and year of diagnosis. Cancer stage was defined according to the American Joint Committee on cancer (AJCC) 7th and 8th edition staging system. Primary outcomes of interest included advanced disease (stage III and IV) at diagnosis, receipt of cancer directed treatment, and achievement of textbook outcome (TO). Cancer-directed treatment included receipt of surgery and chemotherapy. TO is a composite measure of an optimal outcome following surgery, defined as the absence of postoperative complications, extended length of stay, 90-day readmission, and 90-day mortality.^11^ The incidence of postoperative complications was determined using ICD-10 diagnosis codes, as previously described.^12,13^

### Statistical Analysis

The data were presented as medians with interquartile ranges (IQR) for continuous variables and frequencies with percentages for categorical variables. Continuous variables were compared using the Wilcoxon rank-sum test, while categorical variables were assessed using the chi-squared test or Fisher’s exact test, as appropriate. Multivariable logistic regression models were used to examine the relationships between neighborhood trajectories and stage at diagnosis, cancer-directed treatment, and surgical outcomes; odds ratios (OR) and 95% confidence intervals (CI) were reported. Population attributable fractions for each subtheme of SVI were calculated to quantify their contributions to poor surgical outcomes. The models were adjusted for age, sex, Charlson comorbidity index, race, region, stage, site of cancer, year of surgery, insurance, residential area (urban vs. rural) and food environment (food deserts and food swamps). Statistical analyses were conducted using STATA version 17 (StataCorp LLC, College Station). All statistical analyses were two-sided, and a *p* value of less than 0.05 was considered statistically significant.

## Results

### Baseline Patient Characteristics

**Fig. 1:**
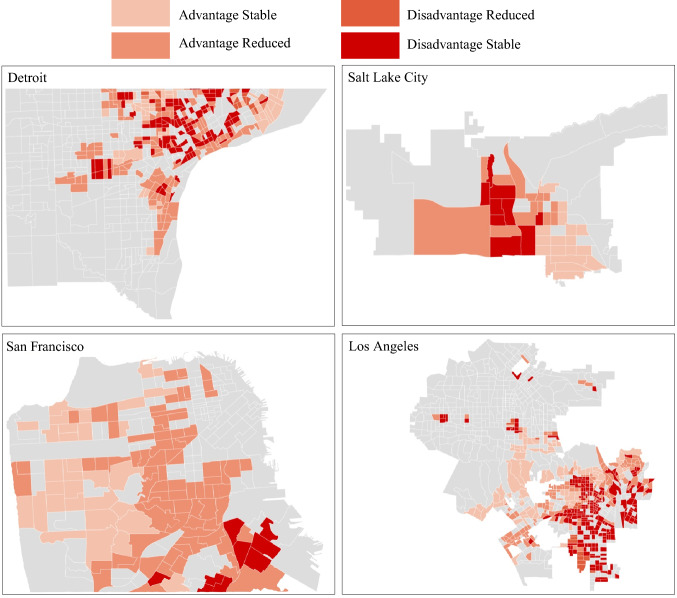
Census tract level distribution of neighborhood trajectories in San Francisco, Los Angeles, Detroit, and Salt Lake City

A total of 15,118 individuals with GI cancer (liver: *n* = 1396, 9.2%; biliary tract: *n* = 1084, 7.2%; pancreas: *n* = 3612, 23.9%; colon: *n* = 7016, 46.4%; rectum: *n* = 2010, 13.3%) were include in the analytic cohort. Median patient age was 78 years (IQR 72–84 years) and most patients were female (*n* = 8375, 55.4%) and had a CCI score of ≤ 2 (*n* = 10,613, 81.2%). Most individuals were white (*n* = 9046; 59.8%), with smaller subsets of Black (*n* = 2744; 18.2%), Hispanic (*n* = 1613; 10.7%), and individuals of other races/ethnicities (*n* = 1715; 11.3%). The majority of individuals resided in the South (*n* = 6477; 42.8%) or West (*n* = 6257; 41.4%), followed by the Midwest (*n* = 2053; 13.6%) and Northeast (*n* = 331; 2.2%) regions of the USA. Stage IV disease (*n* = 5095, 33.7%) was most common, followed by stage II (*n* = 3838, 25.4%), stage III (*n* = 3142, 20.8%), and stage I (*n* = 3043, 20.1%) disease. In terms of neighborhood trajectories, 4608 (30.5%) patients lived in disadvantaged stable census tracts, while 6727 (44.5%) resided in disadvantaged reduced census tracts. Additionally, 448 (2.96%) patients lived in advantaged reduced census tracts, and 3335 (22.1%) lived in advantaged stable census tracts (Fig. 1, Table 1).

**Table 1:** Baseline characteristics of patients stratified by neighborhood trajectory

Characteristics	Total (*n* = 15,118)	Neighborhood trajectory				*p* Value
		Advantaged stable (*n* = 4608, 30.5%)	Disadvantaged reduced (*n* = 6727, 44.5%)	Advantaged reduced (*n* = 448, 2.96%)	Disadvantaged stable (*n* = 3335, 22.1%)	
Age	78 (72–84)	79 (73–85)	78 (72–84)	77 (70.5–84)	77 (71–83)	< 0.001
Sex						0.144
Male	6743 (44.6)	2071 (44.9)	2963 (44.0)	183 (40.9)	1526 (45.8)	
Female	8375 (55.4)	2537 (55.1)	3764 (56.0)	265 (59.1)	1809 (54.2)	
CCI						< 0.001
≤ 2	10,613 (70.2)	3375 (73.2)	4717 (70.1)	291 (65)	2230 (66.9)	
> 2	4505 (29.8)	1233 (26.8)	2,010 (29.9)	157 (35)	1105(33.1)	
Race/ethnicity						< 0.001
White	9046 (59.8)	3517 (76.3)	4531 (67.4)	116 (25.9)	882 (26.4)	
Black	2744 (18.2)	475 (10.3)	840 (12.5)	228 (50.9)	1201 (36)	
Hispanic	1613 (10.7)	247 (5.4)	578 (8.6)	76 (17)	712 (21.3)	
Other	1715 (11.3)	369 (8.0)	778 (11.6)	28 (6.3)	540 (16.2)	
Region						< 0.001
Midwest	2053 (13.6)	414 (9.0)	1005 (14.9)	85 (19.0)	549 (16.5)	
Northeast	331 (2.2)	74 (1.6)	147 (2.9)	24 (5.4)	86 (2.6)	
South	6477 (42.8)	2315 (50.2)	2974 (44.2)	191 (42.6)	997 (29.9)	
West	6257 (41.4)	1805 (39.2)	2601 (38.7)	148 (33.0)	1708 (51.1)	
Minority status						< 0.001
No	9046 (59.8)	3517 (76.3)	4531 (67.4)	116 (25.9)	882 (26.4)	
Yes	6072 (40.2)	1091 (23.7)	2196 (32.6)	332 (74.1)	2453 (73.6)	
Cancer site						< 0.001
Biliary tract	1084 (7.2)	332 (7.2)	465 (6.9)	27 (6.3)	260 (7.8)	
Liver	1396 (9.2)	339 (7.4)	603 (9.0)	41 (9.2)	413 (12.4)	
Pancreas	3612 (23.9)	1198 (26.0)	1611 (23.9)	109 (24.3)	694 (20.8)	
Colon	7016 (46.4)	2153 (46.7)	3108 (46.2)	223 (49.8)	1532 (45.9)	
Rectum	2010 (13.3)	586 (12.7)	940 (14.0)	48 (10.7)	436 (13.1)	
Stage						< 0.001
I	3043 (20.1)	948 (20.6)	1360 (20.2)	102 (22.8)	633 (19.0)	
II	3838 (25.4)	1247 (27.1)	1698 (25.2)	106 (23.7)	787 (23.6)	
III	3142 (20.8)	918 (19.9)	1441 (21.4)	88 (19.6)	695 (20.8)	
IV	5095 (33.7)	1495 (32.4)	2228 (33.1)	152 (33.9)	1220 (36.6)	

### Neighborhood Trajectory and GI Cancer Care

Compared with advantaged stable neighborhoods, disadvantaged stable neighborhoods had a higher number of Black (disadvantaged stable: 36.0% vs. advantaged stable: 10.3%) and Hispanic (disadvantaged stable: 21.3% vs. advantaged stable: 5.4%) residents (both *p* < 0.001). Moreover, patients residing in disadvantaged neighborhoods were younger (disadvantaged stable: 77 years [71–83 years] vs. advantaged stable: 79 years [73–85 years]), had higher CCI score (CCI > 2: disadvantaged stable: 33.1% vs. advantaged stable: 26.8%), and were diagnosed with stage III (disadvantaged stable: 20.8% vs. advantaged stable: 19.9%) or IV (disadvantaged stable: 36.6% vs. advantaged stable: 32.4%) disease (all *p* < 0.001). Moreover, individuals living in disadvantaged neighborhoods were less likely to undergo surgical resection (disadvantaged stable: 55.8% vs. advantaged stable: 59.1%) or receive chemotherapy (disadvantaged stable: 56.7% vs. advantaged stable: 60.3%) (all *p* < 0.001) (Table 2).

**Table 2:** Clinical outcomes stratified by neighborhood trajectory

Characteristics	Total (*n* = 15,118)	Neighborhood trajectory				*p* Value
		Advantaged stable (*n* = 4608, 30.5%)	Disadvantaged reduced (*n* = 6727, 44.5%)	Advantaged reduced (*n* = 448, 2.96%)	Disadvantaged stable (*n* = 3335, 22.1 %)	
Chemotherapy	8845 (58.5)	2777 (60.3)	3927 (58.4)	251 (56.0)	1890 (56.7)	0.009
Surgery	8815 (58.3)	2727 (59.2)	3975 (59.1)	252 (56.3)	1861 (55.8)	0.006
Complications	1773 (23.3)	517 (21.6)	49 (22.6)	780 (22.9)	427 (26.5)	0.004
Extended length of stay	2167 (28.4)	610 (25.5)	610 (25.5)	59 (27.2)	515 (32.0)	< 0.001
Readmission	2175 (28.5)	591 (24.7)	61 (28.1)	1028 (30.2)	495 (30.7)	< 0.001
90-Day mortality	903 (11.8)	229 (9.6)	423 (12.4)	22 (10.1)	229 (14.2)	< 0.001
Textbook outcome	3534 (46.3)	1226 (51.3)	1540 (45.2)	103 (47.5)	665 (41.3)	< 0.001

Following cancer-directed surgery, individuals living in disadvantaged stable neighborhoods were more likely to experience complications (disadvantaged stable: 26.5% vs. advantaged stable: 21.6%) and readmission within 90 days (disadvantaged stable: 30.7% vs. advantaged stable: 24.7%) (both *p* < 0.001). Moreover, individuals residing in disadvantaged stable neighborhoods were more likely to have an extended length of stay (disadvantaged stable: 32.0% vs. advantaged stable: 25.5%) and die within 90 days of surgery (disadvantaged stable: 14.2% vs. advantaged stable: 9.6%) (both *p* < 0.001) (Table 2). On multivariable analysis, after adjusting for baseline characteristics, residents of disadvantaged stable neighborhoods had higher odds of being diagnosed at an advanced stage (stage III and IV: OR 1.29; 95% CI 1.18–1.42) and lower odds of receiving chemotherapy (OR 0.67; 95% CI 0.58–0.76). Moreover, patients living in disadvantaged stable neighborhoods had 32% lower odds of achieving a TO (OR 0.68; 95% CI 0.59–0.77) (Table 3).

**Table 3:** Association between neighborhood trajectory and gastrointestinal cancer care

Trajectory	Advanced disease at diagnosis		Surgery		Chemotherapy		Textbook outcome	
	OR 95% CI	*p* Value	OR 95% CI	*p* Value	OR 95% CI	*p* Value	OR 95%CI	*p* Value
Advantaged stable	Ref		Ref		Ref		Ref	
Disadvantaged reduced	1.12 (1.03–1.21)	0.004	0.90 (0.76–1.06)	0.232	0.84 (0.75–0.94)	0.003	0.80 (0.72–0.89)	< 0.001
Advantaged reduced	1.08 (0.89–1.31)	0.449	0.62 (0.40–0.96)	0.031	0.79 (0.59–1.05)	0.100	0.83 (0.62–1.11)	0.207
Disadvantaged stable	1.29 (1.18–1.42)	< 0.001	0.88 (0.72–1.09)	0.249	0.67 (0.58–0.76)	0.00	0.68 (0.59–0.77)	< 0.001

### Attributable Risk of SVI

**Fig. 2:**
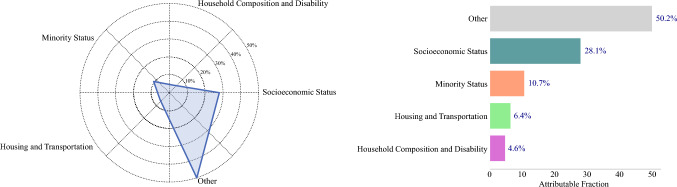
Spider plot illustrating the contribution of each SVI subtheme to poor surgical outcomes

Population attributable fractions for each subtheme of SVI were calculated to quantify their contributions to poor surgical outcomes. SVI was composed of four main subthemes that captured different aspects of vulnerability. Subtheme 1 assessed socioeconomic status, including income, education, and employment, reflecting economic stability. Subtheme 2 examined household composition and disability, focusing on family structures and the presence of disabilities. Subtheme 3 addressed minority status and language, considering racial and ethnic diversity along with language barriers that may hinder access to services. Subtheme 4 evaluated housing and transportation, identifying risks linked to inadequate living conditions and mobility challenges. Among patients who did not achieve a TO, 28.01% were attributable to socioeconomic status, 4.6% to household composition and disability, 10.7% to minority status, and 6.4% to difficulties with housing and transportation (Fig. 2).

## Discussion

While there have been major advancements in cancer care, marked disparities in clinical outcomes persist.^14^ Structural inequity and racism continue to be key drivers of these health inequities; however, effectively capturing and measuring structural inequity remains a challenge.^6^ Notably, individuals residing in socially vulnerable neighborhoods experience delays in surgery, reduced access to follow-up care, and worse overall health outcomes.^7,11,15,16^ Importantly, these neighborhood conditions do not arise in isolation but result from past and present policies, systems, and practices, including historic redlining and more recent gentrification.^17^ To address this gap, the current study utilized trajectory modeling of historical and current socioeconomic status to examine how neighborhood characteristics influenced disease stage at diagnosis, cancer directed treatment, and postoperative outcomes. Individuals living in disadvantaged stable neighborhoods were more likely to be diagnosed with GI cancer at an advanced stage and were less likely to receive chemotherapy compared with individuals in advantaged stable neighborhoods. Furthermore, after surgery, individuals in disadvantaged stable neighborhoods had a higher likelihood of complications, longer hospital stays, readmissions, and mortality within 90 days. The findings of the current study underscore the urgent need for targeted interventions and policies to address structural inequities and promote health equity for patients with GI cancer.

The current study builds on established evidence suggesting a relationship between one’s place of residence and the quality of cancer care received.^18,19^ Previous studies have demonstrated an association between redlining and several SDoH that contribute to disparities in cancer care, such as poor environmental conditions, employment opportunities, and wealth.^20^ Key spatial factors driving inequities in cancer care include socioeconomic disparities and racial/ethnic segregation, both of which independently lead to reduced access to and receipt of care for various medical and surgical conditions.^3,20^ Furthermore, current socioeconomic measures, such as SVI, significantly impact cancer incidence, staging at diagnosis, access to stage-appropriate care, and overall outcomes.^4,14^ This study employs robust statistical methods to evaluate the effects of historical policies and practices of residential redlining in the context of ongoing marginalization and neighborhood development. Notably, individuals residing in disadvantaged neighborhoods, particularly in areas historically affected by redlining and currently facing high social vulnerability, were more likely to receive a diagnosis of GI cancer at an advanced stage. This finding was likely due to health barriers stemming from various structural factors.^20,21^ For instance, limited access to healthcare services can delay screenings and timely interventions.^22,23^ Additionally, lower health literacy may prevent residents from recognizing symptoms or understanding the importance of regular checkups.^24^ Moreover, environmental hazards in these areas, along with lifestyle challenges such as poor nutrition and a lack of safe spaces for physical activity, further exacerbate health risks.^25,26^ Given these findings, it is essential to focus on addressing these disparities to ensure equitable access to screening for all populations.

Interestingly, the receipt of surgery did not differ across neighborhood trajectories. This finding may reflect broader national efforts that have improved access to surgical care, particularly among older adults with Medicare coverage.^27^ However, equitable access to surgery does not necessarily ensure equitable outcomes.^28^ The current study highlights that individuals in disadvantaged stable neighborhoods experienced substantially worse postoperative outcomes, highlighting that disparities persist in the quality and safety of surgical care. These differences likely reflect structural and systemic barriers, including under-resourced healthcare settings, limited access to multidisciplinary support, and broader social determinants that impact recovery and follow-up care.^28,29^ For instance, residents in disadvantaged stable neighborhoods often experience chronic stressors, such as socioeconomic instability, limited access to healthcare resources, and a higher prevalence of comorbidities.^29,30^ These prolonged stressful conditions can negatively impact their overall health status and ability to recover effectively from surgical procedures.^31^ Additionally, disadvantaged stable neighborhoods have a higher number of minority patients, and previous studies have reported that minority-serving hospitals are less likely to provide GI cancer care that complies with National Comprehensive Cancer Network guidelines.^28^ Minority-serving hospitals often serve a disproportionately high number of minority patients and frequently operate under limited resources and financial constraints.^32^ Given the complexity of GI cancer treatment, which requires multidisciplinary and multimodal management, costs are often a significant barrier for lower-resourced hospitals to provide compliant and comprehensive care.^33^ Therefore, the combination of suboptimal care provided at such hospitals and the poor overall health conditions of patients ultimately contributes to worse outcomes among patients living in disadvantaged stable neighborhoods.^30,32^

To address these disparities, there is a need for targeted, multidisciplinary interventions.^34^ The current study quantified the contributions of various subthemes of SVI and highlighted that, among patients who did not achieve a TO, 28.01% were attributable to socioeconomic status, 4.6% to household composition and disability, 10.7% to minority status, and 6.4% to challenges related to housing and transportation. Therefore, it is essential to strengthen primary cancer prevention, improve health literacy, and tackle critical social determinants such as transportation, insurance status, and financial barriers that prevent patients from accessing regular cancer treatment.^21,34,35^ Moreover, policy reforms should focus on dismantling the legacy of redlining by advocating for equitable housing opportunities and increasing funding for healthcare facilities in underserved neighborhoods.^7^ Increasing accessibility to healthcare can be achieved through mobile clinics and enhanced telehealth services, ensuring that screenings and preventive care reach those in need.^36^ Moreover, community outreach programs will improve health education and empower residents to recognize symptoms and understand the importance of regular checkups.^37^ Providing financial and technical support to minority-serving hospitals will improve the quality of care they offer.^32^ By fostering partnerships among healthcare providers, community organizations, and local governments, we can create initiatives that address social determinants of health.^32,35^

The current study should be interpreted considering several limitations. The use of a large national database in the study was a notable strength. However, the retrospective design and dependence on an administrative database introduced the potential for residual confounding bias. Additionally, SEER database lacked granular data on patient and hospital characteristics, such as distance traveled for care and hospital Commission on Cancer accreditation. The study sample consisted of patients 65 years of age or older with Medicare coverage, which may have limited the generalizability of the results to younger patients and those with private insurance. SEER data have low sensitivity for detecting the receipt of chemotherapy. For example, previous research has noted that the overall sensitivity of SEER data in identifying chemotherapy is 68%.^38,39^ Additionally, the database has limitations in capturing chemotherapy receipt, including incomplete documentation of treatment details and potential biases from unmeasured factors that may influence treatment decisions. The current study utilized the Social Vulnerability Index (SVI) to identify factors contributing to poor outcomes among individuals residing in historically redlined neighborhoods. While SVI is a validated and comprehensive measure of social determinants of health, it is notable that approximately 50% of the population-attributable risk for not achieving a TO was not explained by its subthemes. This residual risk likely reflects unmeasured factors, and future studies should incorporate additional variables that capture these complex, multidimensional influences on surgical outcomes.

In conclusion, individuals living in disadvantaged stable neighborhoods are more likely to be diagnosed with GI cancer at a more advanced stage, are less likely to receive chemotherapy, and experience poorer surgical outcomes. There is an urgent need for targeted interventions and policies that tackle critical social determinants of health such as transportation, insurance status, and financial barriers to ensure health equity for patients with GI cancer.
